# The Influence of Person-Environment Fit on the Turnover Intention of Nurses in Jordan: The Moderating Effect of Psychological Empowerment

**DOI:** 10.1155/2021/6688603

**Published:** 2021-03-16

**Authors:** Saleh Amarneh, Ali Raza, Sheema Matloob, Raed Khamis Alharbi, Munir A. Abbasi

**Affiliations:** ^1^Graduate School of Business, Universiti Sains Malaysia, Penang 11800, Malaysia; ^2^Department of Business Administration, Sukkur IBA University, Sukkur 65200, Pakistan; ^3^School of Management, Universiti Sains Malaysia, Penang 11800, Malaysia; ^4^College of Administration and Financial Science, Taif University, Taif 26571, Saudi Arabia; ^5^Benazir School of Business, Bhutto Shaheed University Lyari, Karachi 74660, Pakistan

## Abstract

There is an acute shortage of nurses worldwide, including in Jordan. The nursing shortage is considered to be a crucial and complex challenge across healthcare systems and has stretched to a warning threshold. High turnover among nurses in Jordan is an enduring problem and is believed to be the foremost cause of the nurse shortage. The purpose of this study was to investigate the multidimensional impact of the person-environment (P-E) fit on the job satisfaction (JS) and turnover intention (TI) of registered nurses. The moderating effect of psychological empowerment (PE) on the relationship between JS and TI was also investigated. Based on a quantitative research design, data were collected purposively from 383 registered nurses working at private Jordanian hospitals through self-administered structured questionnaires. Statistical Package for Social Sciences (SPSS) 25 and Smart Partial Least Squares (PLS) 3.2.8 were used to analyze the statistical data. The results showed that there is a significant relationship between person-job fit (P-J fit), person-supervisor fit (P-S fit), and JS. However, this study found an insignificant relationship between person-organization fit (P-O fit) and JS. Moreover, PE was also significantly moderate between JS and TI of nurses. This study offers an important policy intervention that helps healthcare organizations to understand the enduring issue of nurse turnover. Additionally, policy recommendations to mitigate nurse turnover in Jordan are outlined.

## 1. Introduction

Nurses make up the largest section of healthcare professionals, and it is estimated that approximately 90% of direct patient care is provided by nurses [1]. This profession offers more than simply performing duty; instead, it requires self-sacrifice [2] and empathy [3]. It is estimated that there are 29 million nurses and midwives across the world, comprising approximately half of the global healthcare workforce [4, 5]. The nature of nursing requires the potential to understand the needs of others [6], which, in particular, positively affects patient care and the overall quality of the health delivery system. Despite nurses' critical and irreplaceable responsibilities in overall healthcare, the shortage of qualified nurses is a global concern. According to the World Health Organization, there is shortage of more than 9 million nurses, and this number is continuously rising [5], potentially threatening patient outcomes and compromising the overall health delivery system [7]. Specifically, research shows that approximately 4%–54% of nurses across the world intend to leave the profession [8], raising an important concern over healthcare organization practices that will potentially lead toward negative patient outcomes [9]. In this situation, it is imperative to take precautionary measures to prevent nurses from resigning from their professions [10]. Therefore, minimizing turnover is a priority for healthcare organizations who are concerned about the survival of their entity, particularly with the current escalating nursing shortage [11].

The epidemic nature of nurse shortage is particularly affecting the Jordanian health sector. The Jordanian health sector is considered one of the most developed and modern healthcare systems in the Middle East region. The sector is equipped with the latest machinery used by internationally qualified and world-class doctors working in internationally accredited hospitals [12]. However, despite the attractiveness of the health sector [13], the high turnover of nurses is an enduring problem and is considered the foremost cause of the nurse shortage in Jordan [14]. Previous research [15, 16] extensively used the study of Hayajneh et al. [17] to address the nurse turnover in Jordan. This study indicated that the overall turnover rate among registered nurses in Jordanian hospitals was 36.6% in 2009. However, there are no data available stating the current turnover rate. Therefore, based on the latest statistics provided by the Jordanian Ministry of Health in 2019 [18], we calculated and found that there is a shortage of approximately 50,164 (61%) nurses in the country's healthcare system, whereas only 31,822 nurses are registered and assumed to be working in the hospitals. Furthermore, it is estimated that 81,976 nurses are required to cater to the needs of a population of over 10 million in the country. These figures show that Jordan's healthcare system is hurtling toward a severe nurse shortage. It is important to mention that Jordan is at risk of facing difficulties due to its increasing population that is expected to double by 2030, an increase in the prevalence of noncommunicable diseases (NCDs), and already existing Human Resource for Health (HRH) challenges such as retention and continuous training [19]. In addition, extensive research has revealed a link between nursing staff turnover and patient outcomes in terms of patient health [20], length of stay in the hospital [21], and quality of care [22]. In addition to the potential risk regarding the health of the general public, nurse turnover remains a serious and costly concern for most healthcare organizations. The high turnover among nursing staff severely impacts healthcare organizations in terms of substantial financial and nonfinancial costs [23], which is worrisome and needs immediate attention.

Although there are various factors that can influence one's decision to quit job [24], previous studies have shown the research on nurse turnover to be related to motivation [25], healthcare organizational climates [26], nurses wages [27], healthcare organizational characteristics [28], and coworker support for nurses with children [29]. Besides, Hayajneh et al. [17] determined the rate of nurse turnover in Jordanian hospitals to be 36.6% in 2009 and also identified that the intention of nurses was influenced by geographical regions, healthcare systems, and places of residence. Based on Hayajneh et al.'s [17] research, it is needed to carry out further investigation to examine the phenomena in broader sense.

With respect to the shortage of nurses, Neisner and Raymond [30] indicated unsatisfactory or low JS as a determinant factor. Similarly, Newman et al. [31] pointed out that nurses' satisfaction is a key factor for their retention, while dissatisfaction, in general, is the most important causal factor of nurses leaving practice. This dissatisfaction, and the resultant abandonment of nursing practice, is mainly determined by poor management [31]. In this way, research has shown that one of the important reasons behind nurses' intent to leave is their incompatibility or mismatch with the healthcare organization environment, which is termed P-E fit [32]. In other words, the fit between a person and the environment in which they work results in positive outcomes (e.g., JS), while a lack of a fit produces psychological, physiological, and behavioral strains (e.g., dissatisfaction and burnout) [32, 33]. As a result, the employee may decide to leave the workplace as a final step of withdrawal behavior. Current research is also attempting to study the moderating effects of PE on the TI of nurses. PE is considered a motivational orientation that comprises four cognitions (i.e., meaning, competence, autonomy, and impact) that reflect the feelings of individuals, i.e., the motivation and competency to actively achieve work expectations [34]. Combined, the four cognitions imply that employees find their work meaningful, they feel competent to perform work-related tasks, they feel that they have adequate autonomy at work, and they have belief that their actions can influence their work environment in a positive manner [34]. In addition, PE substantiates the positive influence on P-E fit perceptions, which, in return, restores the satisfaction level of individuals at work [35]. In the nursing profession, workplaces embedded with empowerment yield positive workplace behaviors and attitudes that are consistently linked to the retention of nurses (e.g., JS) [36]. Furthermore, the study of Greco et al. [37] validated the concept that when nurses feel empowered, they are more likely to experience and attain the fit between their expectations and the healthcare organization in which they work.

To better understand and prevent turnover, this study aimed to investigate (i) the main effects of P-E fit on the JS and TI of nurses and (ii) the moderating role of PE on the relationship between the JS and TI of nurses in Amman, Jordan.

## 2. Theoretical Background and Hypothesis Development

The management literature clearly shows that growing attention is being paid to the concept of P-E fit since it offers many insights into the link between an organization's policies and activities and the attitude and behavior of its employees [38]. Relying on P-E fit theory, organizations and their representatives have a fundamental concern regarding how well their individual employees' characteristics and the organization's environment suit each other. Organizations want to seek out people who will best meet the job requirements, adapt to professional development, change job requirements, and stay loyal and committed to the organization. Meanwhile, prospective employees want to find organizations that harness their specific skills and meet their specific needs [39]. Fit is recognized by comparing the internal aspects of a person, such as their values, personality, goals, and abilities, to conceptually related external environmental elements, such as the organization's or supervisor's values, personality, goals, and work requirements [40]. Ultimately, a key focus in virtually every P-E fit theory is that a better fit will lead to superior results, such as higher JS, better work transition, higher job performance, less stress, greater career achievements, and a greater likelihood of retention [41], as well as less TI [42]. Unfortunately, less research has focused on the possible intervening variables that may help to explain how the compatibility between a person and his/her corresponding environment (e.g., organization, job, and supervisor) comes to impact his/her attitudinal and behavioral outcomes.

**Figure 1:**
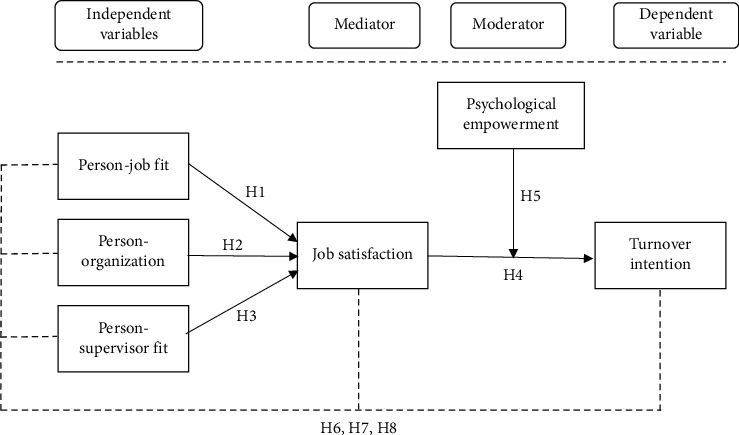
Research framework.

Our proposed model emphasizes an examination of the nexus among the three dimensions of P-E fit, JS, and TI, as described in Figure 1. This research employed three types of fit—i.e., the compatibility of a person with his/her job, organization, and supervisor—to form the P-E fit as these dimensions have emerged as essential research fields [32, 40, 43]. To the best of the authors' knowledge, there is very scarce research about P-E fit—or any form of fit—in a Jordanian context. However, given the evidence stating that various forms of P-E fit have a unique impact on the result obtained [44], this study contributes to this knowledge by validating the P-E fit in Jordan, particularly in healthcare organizations that are operated privately. Simultaneously, this study contributes to the existing body of the literature in managing healthcare professionals by examining the moderating role of PE between JS and TI.

### 2.1. Person-Job (P-J) Fit and Job Satisfaction

P-J fit is characterized as the correspondence between a person's abilities and the demands of a job, or a person's needs/wishes and what a job provides [45]. That is, P-J fit is related to an individual's compatibility with an exact job [42]. In other words, it shows the degree of association between employees' skills and job tasks. Research has uncovered that JS drives many advantages for both employees and the organization [46]. The quality of service and the retention of key workers can be related to workplaces in which staff can achieve a sense of satisfaction [47]. Prior investigations have proclaimed a relationship between P-J fit and JS using multiple contextual settings [48, 49]. For instance, Lauver and Kristof-Brown's [42] investigation concluded that P-J fit has a unique impact on JS. Moreover, evidence from an Eastern context was provided about the existence of the relationship between P-J fit and JS, having studied university employees in Pakistan [50]. Based on the above substantive review, the following hypothesis was deduced.

**Hypothesis 1 .**

(H1). There is a positive relationship between person-job fit and job satisfaction.

### 2.2. Person-Organization (P-O) Fit and Job Satisfaction

P-O fit is amongst the most widely researched forms of P-E fit in the Human Resource Management (HRM) domain. P-O fit stimulates communication between employees, promotes employee identification with an organization, creates an environment of confidence, and encourages favorable attitudes and behaviors related to work [51, 52]. From the perspective of the organization, it is the means to retaining adaptable and loyal employees, which is crucial in a competitive business environment and a tight labor marketplace [43]. Meanwhile, P-O fit from an employee's point of view may be a means to display the level of fulfilment of their desires and expectations [53]. Kristof [54] described P-O fit as the compatibility between individuals and organizations that occurs when (1) at least one of the two parties offers what the other requires, (2) they share the same essential features, or (3) both. Compatibility may also encompass other factors such as ideals, attitudes, features, personality, or objectives [55]. In view of this, Kilroy et al. [55] proclaimed that the most correlated and effective predictor of employee outcomes is the uniformity between an individual's personal values and those of the organization, often known as “value congruence.” Accordingly, it is reasonable to consider that P-O fit is the main cause of JS [56], leading to the following hypothesis.

**Hypothesis 2 .**

(H2). There is a positive relationship between person-organization fit and job satisfaction.

### 2.3. Person-Supervisor (P-S) Fit and Job Satisfaction

Another emerging dimension of P-E fit is P-S fit—the “fit between (an) employee and his or her direct supervisor characteristics.” [44] One significant factor sourced from the P-E fit theory is value congruence within the P-S fit construct [57]. Currently, in terms of the diversity of social norms in the workplace, choosing a supervisor who can cope with this diversity is very critical. Meanwhile, having a supervisor with similar values can offer a stronger sense of fit, thereby improving employees' satisfaction [58]. Moreover, the compatibility between employees and their supervisor will facilitate the employee interaction with the surrounding “organization,” [58] which may indirectly impact an individual's fit with their organization. In contrast, Van Vianen et al. [59] uncovered that supervisors and organizations may serve as fairly independent sources of reference to determine the fit of a person. This idea was upheld by a study that autonomously researched the impact of P-O and P-S on JS [41], which also supported the positive role of P-S fit in JS. The following hypothesis was deduced on the basis of the above substantive review.

**Hypothesis 3 .**

(H3). There is a positive relationship between person-supervisor fit and job satisfaction.

### 2.4. Job Satisfaction and Turnover Intention

JS, a concept that is widely studied in organizational behavior research, is commonly conceptualized as one's feelings or state of mind toward the nature of their work [60]. In simpler terms, JS is “the extent to which people like their jobs.” [61] The research witnessed that JS has a stable and enormous connection with positive work outcomes. For example, when employees enjoy their work, they become more efficient and remain motivated which ultimately increase the service quality [62]. In addition, JS contributes to cost reduction in terms of less absence, work errors, and turnovers which are a noteworthy outcome. The employees who are satisfied with their jobs tend to be in their positions for longer time, whereas the dissatisfied ones possess higher level of intention to leave their workplace [63]. Based on the extensive research, we can reasonably assume that less JS may cause the TI.

**Hypothesis 4 .**

(H4). There is a negative relationship between job satisfaction and turnover intention.

### 2.5. Psychological Empowerment

Spreitzer [34] defines PE as motivational orientation consisting of four cognitions (meaning, competence, autonomy, and impact). PE can be depicted as an individual's perspective of his or her job role and capability to influence work-related outcomes [64, 65]. For instance, empowered employees enhance their performance by rapidly recovering from service delivery mistakes, enriching their knowledge from these recoveries, and creating and redesigning approaches and materials [66]. The literature shows considerable evidence that PE has a significant positive influence on various work-related outcomes such as increased organizational commitment [67, 68], innovative behavior [34, 69], job satisfaction, and performance [61, 62]. Based on the above positive related outcomes of PE, we used PE as a moderator between the relationship of JS and TI. We argued that nurses need to feel psychologically empowered, and when they are, they feel valued in the healthcare organizations, which keeps them satisfied and retained in the workplace. On the basis of the above argument, the following hypothesis is proposed.

**Hypothesis 5 .**

(H5). Psychological empowerment moderates the effect of job satisfaction on turnover intention.

### 2.6. The Mediating Effect of Job Satisfaction

There is a general understanding that P-J fit has major consequences on individual behaviors and work outcomes [49]. Employee satisfaction is strongly influenced by employees' assessments of the work and tasks they carry out, which are essential elements of the P-J fit [42]. Low levels of JS are viewed as an enormous reason for employee turnover in the medical services industry [70]. As a causal relationship, Chhabra [71] found that decreases in P-J fit, which resulted in decreases in JS, were more likely to contribute to higher TI. Accordingly, the following hypothesis was deduced.

**Hypothesis 6 .**

(H6). Job satisfaction mediates the link between person-job fit and turnover intention.

P-O fit is often conceptualized as a supplementary fit (i.e., matching the organization). In other words, P-O fit relies in the exchange relationship between a person and his/her organization. This exchange relationship can be seen by the organization as a “demands-abilities fit” happens when an employee's capabilities satisfy the demands of the organization. Meanwhile, the employee sees this relationship as “needs-supplies fit,” which emphasizes the requirements and desires of the employee to be fulfilled by the organization [49]. In this sense, those with a high P-O fit will prosper more effortlessly compared to those with a low P-O fit because they can allocate and invest their resources in order to obtain more resources and thus maximize their fit within their environment [52]. Previous investigations have proclaimed that P-O fit leads to desirable employee outcomes (e.g., attitudinal outcomes); for instance, a positive role of P-O fit in evoking employees' JS was found by investigating employees from Eastern and Western contexts [72, 73]. Recently, an investigation by Jin et al. [52] uncovered a causal chain from the input (P-O fit) to attitudes (JS) and TI (employee behavior). Accordingly, the following hypothesis was deduced.

**Hypothesis 7 .**

(H7). Job satisfaction mediates the link between person-organization fit and turnover intention.

Employees do not perform in a social vacuum, but rather depend on others with whom they interact, especially their supervisor [74]. Employees regard supervisors as representatives of the organization, and supervisors' behaviors are therefore expected to reflect the organizational culture [40]. Meanwhile, supervisors are able to evaluate the alignment of employees' performance to the organization's values and goals [74]. P-S fit is commonly referred to as congruence or resemblance between an individual's and their supervisor's characteristics [40]. The compatibility between employees and their supervisor will increase the satisfaction level [58]. JS is considered to be a key issue for healthcare professionals all over the world [75]. Research among the nurses working in the U.K.'s National Health Service has shown that JS is negatively associated with TI [76]. Accordingly, the following hypothesis was deduced.

**Hypothesis 8 .**

(H8). Job satisfaction mediates the link between person-supervisor fit and turnover intention.

## 3. Materials and Methods

### 3.1. Study Setting and Sample

A quantitative, cross-sectional approach was adopted to conduct this study. A survey technique was used to collect the data from the nursing staff of private hospitals in Amman Governorate, Jordan's capital. Amman city has 43 hospitals that represents 67% of the private hospitals in Jordan. Prior to data collection, official permission was obtained. The data were gathered through self-administered structured questionnaires through purposive sampling. Prior to filling in the questionnaires, the nurses were requested to read the cover letter and to sign the consent form, which ensured them about the confidentiality of their responses. In total, 600 questionnaires were disseminated to the respective hospitals, of which 383 (63.8%) were filled in and returned to the researchers. Ethical approval for this research was obtained from the Research Ethics Committee of the Jordanian Ministry of Health under the approval code MOH/REC/2019/192.

The means, standard deviations, correlations, and reliabilities among the study variables are presented in Table 1. The Pearson correlations among the variables ranged from 0.21 to 0.66 (*p* < 0.01) which demonstrates an adequate level of reliability for the further tests.

Given the exploratory nature of the study, SPSS 25 was used to analyze the participants' demographic profiles, while hypothesis testing of the model was performed using SmartPLS version 3.2.8. The measurement model was tested in the first step to validate the questionnaire, and the structural model was tested in the second step to evaluate the hypotheses [77].  Table 2 shows the demographic profiles of the participants.

The sample of participants consisted of 227 (65%) female and 122 (35%) male nurses, of which 54% were married and 46% were single. More than half of the participants were aged between 30 and 39 years (51.1%), and almost 30% of the nurses were aged ≥40. In terms of work experience in their current healthcare organizations, 44.2% of the participants had been there for 1–5 years, 17.9% between 6 and 10 years, 17.2% between 11 and 15 years, and only 20.7% more than 15 years.

### 3.2. Measures and Variables

To assess TI, the six-item Turnover Intention Inventory Scale (TIIS-6) validated by Bothma and Roodt [78] was used. The content of TI included the intention to quit in the future and finding a new opportunity in the current market. The P-J fit was measured using the three-item scale of Cable and DeRue [79] for the needs-supplies fit. A measure of the P-O fit was adopted from Alniaçik et al. [80]. The scale consisted of four items and was slightly modified to match the context of hospitals by changing the word “organization” to “healthcare organization.” To assess P-S fit perceptions, we used the three-item P-O fit perception scale developed by Cable and DeRue [79] and then adapted by Zhang et al. [81] to measure the P-S fit. To measure JS, a total of six items were adopted for this study, developed by Zeffane and Bani Melhem [82] from their original source [83]. An average summed score was calculated, with a higher score indicating higher JS. PE was measured with Spreitzer's [34] 12-item scale divided into four dimensions of three items each: meaning, competence, self-determination, and impact. In Hancer et al.'s [84] study, these scales were slightly modified and used for measuring the PE of service employees. In this paper, we used Hancer et al.'s [84] unidimensional version for the measurement of PE. Participants were asked to rate all of the items of the scale on a five-point Likert scale, ranging from 1 (strongly disagree) to 5 (strongly agree).

## 4. Results

### 4.1. Measurement Model

Structural equation model (SEM) was used to test the proposed hypothesis. In this way, Smart PLS-SEM is one of the most appropriate and widely used methods to obtain the results for measurement and structural models [85]. It is based on two major steps for measuring a research model, i.e., reliability and validity of the research model. Reliability was tested through factor loadings, composite reliability (CR), and average variance extracted (AVE). In the present study, items with less than 0.5 factor loading were removed [86]. However, all of the measured CR (0.7) and AVE (0.5) values were acceptable.

Table 3 shows the factor loadings, CR, and AVE values, all of which were greater than the threshold values.

Similarly, the discriminant validity is shown in Table 4 through the heterotrait-monotrait ratio.

**Figure 2:**
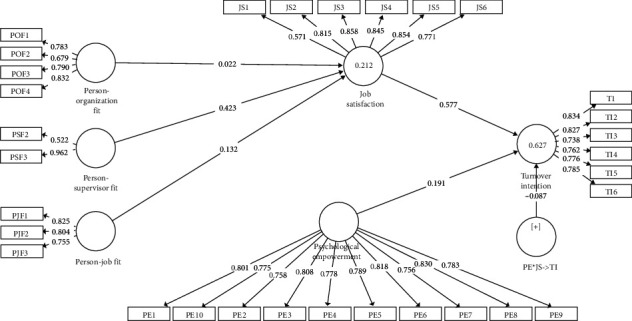
Measurement model.

The measurement model of the current study is detailed in Figure 2.

Figure 2 reports two important observations that have a great significance on measuring the reliability of the latent variables, including the CR, AVE, and factor loadings. As per the rule of thumb, factor loadings should be greater than 0.50 [86]. In the present study, all of the loaded items greater than 0.5 for P-O fit (POF), P-S fit (PSF), P-J fit (PJF), job satisfaction (JS), psychological empowerment (PE), and turnover intention (TI) were acceptable. In addition, the item loadings forming the AVE should be greater than 0.5 [86]. In our study, the POF (0.598 > 0.50), PSF (0.599 > 0.50), PJF (0.632 > 0.50), JS (0.628 > 0.50), PE (0.624 > 0.50), and TI (0.621 > 0.50) were acceptable; thus, the measurement model was valid.

### 4.2. Structural Model

The results of the hypothesis are shown in Table 5.

Similarly, Table 6 reports the results of the indirect hypotheses.

Table 5 shows the *t*-values and path coefficients found for the dimensions of P-E fit as the independent variable and JS as the dependent variable. In addition, JS is the independent variable with TI being the dependent variable. In the present study, two out of three of the hypotheses were supported: P-J fit (*t* = 2.743; *β* = 0.132) and P-S fit (*t* = 8.180; *β* = 0.423) are positively related to JS. Meanwhile, P-O fit (*t* = 0.467; *β* = 0.022) was found to be insignificantly related to JS. Similarly, JS (*t* = 6.234; *β* = 0.577) is statistically negatively related to TI. In addition, PE had a significantly moderate relationship between JS and TI (*t* = 2.478; *β* = 0.570) among nurses in Jordan. These results reveal that the nurses in private healthcare organizations experience high PE, resulting in more satisfaction with their job and a reduction in TI due to psychological interventions. However, when the nurses experience low PE, they tend to be less satisfied with their jobs, leading to higher TI.

**Figure 3:**
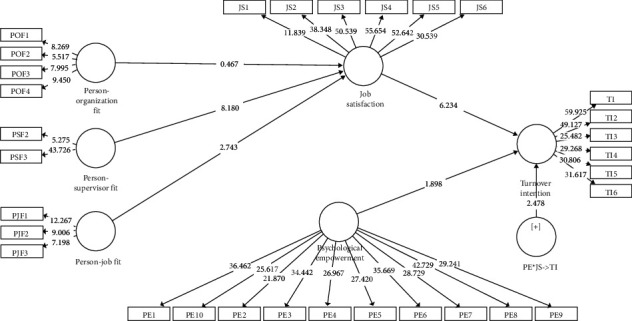
Structural model.

To determine whether JS indirectly mediates the relationship between the dimensions of P-E fit and TI, two-tailed results were generated by SmartPLS. Table 6 shows the indirect effect of JS on TI. It is postulated that JS is able to mediate a positive relationship between P-J fit and TI (*β* = 0.076; *t* = 2.38) and P-S fit and TI (*β* = 0.244; *t* = 5.333). In addition, JS does not mediate the relationship between P-O fit and TI (*β* = 0.013; *t* = 0.448). Therefore, Hypotheses 2 and 7 could not be accepted, while Hypothesis 5 could not be rejected. The aforementioned results are presented in Figure 3.

**Figure 4:**
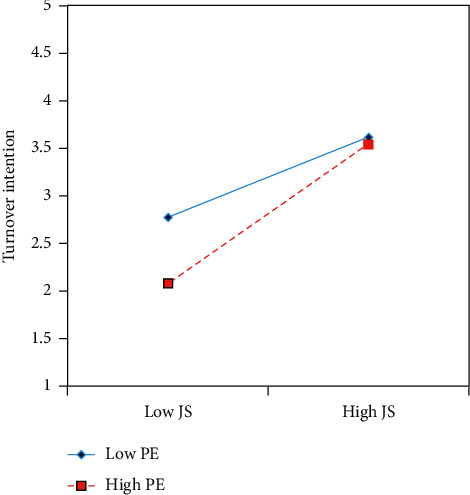
Interaction effect of job satisfaction and psychological empowerment on turnover intention.

In addition, the current study found that PE weakens the negative effect of JS on TI (Figure 4). It was also shown that even if less-satisfied employees have a high level of PE, they will have a lower tendency to leave his/her healthcare organization.

Interestingly, the results revealed that PE has a significant moderating effect on the relationship between JS and TI. Thus, it was proven that those nurses who have strong PE are less likely to quit their job. The findings of this study endorse that JS can divert the intentions of nurses from leaving their jobs.

## 5. Discussion

Driven by P-E fit theory, the present study provided a novel insight into the constructs of and contributions to the TI of nurses. The current research contributes to findings regarding the identification of the relationship between the individual, i.e., nurses, and the organization, i.e., healthcare organizations, finding that it is substantially related to the process of TI. Therefore, the purpose of this study was to deepen our understanding of the role of the three dimensions of the P-E fit (i.e., P-J fit, P-S fit, and P-O fit) in the JS and TI of the nurses working in the hospitals of Amman, Jordan.

The findings indicated that P-J fit is significantly associated with nurses' JS. P-J fit refers to the ability of an employee to complete a specified job that matches the actual requirements of the job or to the match between an individual's wishes and needs and the characteristics of the job [87]. These results also seem to be consistent with a recent study [88] that affirmed that employees are satisfied and stay tuned into an organization when they believe that their jobs are in accordance with their knowledge, skills, and abilities. This belief may create harmony between the employee and the workplace, specifically with their jobs. In addition, Edwards [45] revealed that a high level of fit between a person and their job leads to high motivation in said person. Such individuals may have considerably increased performance in work, overall satisfaction, and attendance. Furthermore, P-J fit also initiates individuals to perform better in teams and to produce meaningful work [89]. Furthermore, we also found that the better the P-J fit is, the less likely the nurses were to quit jobs and the more likely to retain at their workplace. This result is consistent with a previous investigation [90].

Similar to previous findings, the present study also found a significant relationship between P-S fit and nurses' JS. These findings are consistent with a recent investigation, Andela and van der Doef [91], which confirmed that an appropriate match between an individual and their job and supervisors yields satisfaction at work, reduces burnout, and lessens the intention to leave their job. These findings also confirm the results of Chuang et al. [92], who showed a significant relationship among the P-E fit dimensions, JS, and TI. Moreover, research has shown that the role of nurses' supervisors significantly interacts with their JS and leads to an improved quality of patient care [93].

Contradictory with previous findings, this study found an insignificant relationship between P-O fit and JS. These findings are consistent with previous studies, i.e., [68, 94], which concluded that nurses distrust healthcare organizations' policies when they see a discrepancy in their common values and those of the host organization. This indicates that the individual goals and personal desires of nurses are not in tandem with the policies and culture of health organizations. The results of current research are in disagreement with previous studies that showed that when nurses perceive P-O fit, this has a positive impact on JS [91, 95]. It is also important to mention that individual P-O fit perceptions may change over a period of time during a nurse's tenure in a health organization [96]. Moreover, the study in the USA also found that a lack of a P-O fit can lead to decreased JS and increased TI [97]. Similarly, the study of Brown and Yoshioka [90] also witnessed that a better P-O fit decreases the intention to quit among employees.

In addition, the current study found that JS has a negative and significant impact on the TI of nurses. Evidence was found for the indirect effects of two out of the three P-E fit elements on TI through JS. It was only P-O fit that did not exert its effects on TI, neither directly nor indirectly via JS. In this way, JS was found to be a total mediator of the effects of P-J fit and P-S fit on TI. One possible justification is the age of the participants, the majority of whom were less than 40. These nurses could have been more likely to consider leaving their jobs in an attempt to achieve career advancement in a better organization or place. These findings are complementary and could be explained by the fact that as a healthcare worker gets older, they may become more adapted to their work and less ambitious. In contrast, younger workers are more active, problem-focused, and reactive to work strains and may have high ambitions to pursue wealth and status. The current findings suggest that nursing leaders should focus on cultivating nurses' values and improving their departments' culture. The high risk, workload, and pressure presented by nursing may leave nurses with insufficient time and energy to actively participate in organizational management and decision-making, which diminishes their perceptions of their impact [98].

This study found that PE weakens the negative and significant relationship between JS and TI. Nurses need to attain PE to reduce their TI. Our findings suggest that nurses tend to hold intentions to resign from their positions and eventually go on to quit their jobs, which could potentially exacerbate the nursing shortage. However, such TI may be reduced if nurses experience positive PE and confidence in their work role [99]. The results of this study support the concept that psychologically empowered employees will feel more empowered and that they will perceive higher autonomy to take decisions [100]. Empowered employees bring novel ideas to the organization [101]. In addition, the findings are interesting in light of previous research [102, 103] that suggests that psychologically empowered employees feel that their tasks are meaningful and intended to achieve the organizational objectives [102]. Furthermore, nurses may feel that that they are competent to perform their assigned tasks [69]; are confident that they can complete their assigned tasks [103]; and their work has a significant impact on the overall healthcare organizational objectives [100]. Consequently, nurses experiencing such a work environment seem to have greater retention in a healthcare organization.

## 6. Policy Interventions

The study has implications for nurses' leaders and healthcare organizations that how they preserve their nurse personnel satisfaction and retain them in the workplace. To address this issue, one effective way would be for the nurse's leaders and healthcare organizations to think carefully and honestly about their organizational values. In other words, the healthcare organizations should honestly articulate their values and overcome the potential conflicts through the dialogue. The congruence of nurses with the healthcare organization positively impacts individual productivity (i.e., patient care) and the overall quality of the service provided [68, 94]. Similarly, ambiguous values may lead towards the value incongruence as the P-O fit relationship may not be understood well enough to be articulated, thereby not being effectively addressed. To address these issues, nurses' leaders and healthcare organizations need not only to carefully recognize and align their mission and values in the hiring process but also make the applicants informed at the entry stage to avoid possible mismatch. Specifically, to combat the high turnover of nurses, it is important to note that the perceptions of applicants of their fit with a healthcare organization are a predictor of their job choice [32]. Therefore, the healthcare organization should provide an honest presentation of the workplace values, as well as the expectations of the work environment prior to taking nurses on board.

In addition, job demand includes shift duty timings, and related work protocols need to be carefully designed so that work must not be conflicted with nurses' personal and familial roles. In this context, clearly articulated values in the organizations help to attract and retain a homogenous workforce [104]. In the context of the current study, it seems that the surveyed nurses experienced value conflicts after entering the workforce. In this context, the study of Duchscher [105] revealed that the proper orientation programs during recruitment can prevent “transition shock” for new nurses.

For healthcare organizations, it is important to understand that improved retention leads to improved patient care, uplifts patient satisfaction, and reduces patient length of stay in the hospital [106], as well as financial benefits. For example, a previous estimate showed that it costs in excess of 150% of a nurse's annual salary to recruit, select, and train a replacement [107]. Healthcare organizations that improve retention could hence reap considerable financial benefits in a time of increasing budgetary constraints. In this context, this study offers PE as an effective tool to restore the satisfaction of nurses and to help them retain their positions in hospitals. In doing so, healthcare organizations should seek to impact nurses' JS with a practice that defines “empowerment over quality job results.” The estimates show that JS is 2.23 times higher in hospitals where nurses feel encouraged after a failure (1.68 for PE over quality job results). Therefore, it is crucial to consider PE over job results, the influence of which also increases over time (age), suggesting that this strategy is also relevant for junior nurses. As such, healthcare organizations should tailor their human resource strategies in way that aligns their goals while keeping nurses satisfied at the workplaces.

## 7. Study Limitations

This study used a cross-sectional research design, which raises questions about causality. Our research was based on the logic that nurses usually form P-E fit during the employment period [108], but it is equally plausible that they can form P-E fit after a period of employment. Additionally, our data were collected from private hospitals in Amman, Jordan. Therefore, we are uncertain about the extent to which the findings may be generalized to nurses in the public healthcare sector. The turnover intention of the nurses can be examined by other potentially related variables such as emotional labor [109], role conflict [110], resilience [111], workplace violence [112], need for achievement, and work-life conflict [113]. In addition, findings could be enriched by adopting qualitative research design in which surveyed nurses could be interviewed to deepen our understanding of TI with studied variables.

## 8. Conclusion

The current study investigated the influence of P-E fit on the TI of nurses in Jordon with the moderating effect of PE. The results showed that there is a significant relationship between person-job fit (P-J fit), person-supervisor fit (P-S fit), and JS. However, this study found an insignificant relationship between person-organization fit (P-O fit) and JS. Moreover, PE was also significantly moderate between JS and TI of nurses. Based on the results, the policy intervention is also outlined to mitigate the nursing turnover issue in Jordon.

## Figures and Tables

**Table 1:** Summary of means, standard deviations, and correlations of all variables.

	Variables	M	S	1	2	3	4	5	6
1	Person-job fit	3.46	0.63	—					
2	Person-organization fit	3.74	0.66	0.66^*∗∗*^	—				
3	Person-supervisor fit	4.27	0.64	0.27^*∗∗*^	0.45^*∗∗*^	—			
4	Person-supervisor fit	4.01	0.52	0.21^*∗∗*^	0.39^*∗∗*^	0.21^*∗∗*^	—		
5	Turnover intention	3.82	0.46	0.28^*∗∗*^	0.34^*∗∗*^	0.26^*∗∗*^	0.44^*∗∗*^	—	
6	Psychological empowerment	3.77	0.39	0.31^*∗∗*^	0.29^*∗∗*^	0.31^*∗∗*^	0.34^*∗∗*^	0.23^*∗∗*^	—

Notes: *∗∗*, *p* < 0.01(two-tailed test); *N* = 349, the values on the diagonal.

**Table 2:** Demographic profiles of the participants (*N* = 349).

Characteristics	Range/category	Frequency (%)
Age (years)	20–29	66 (19)
	30–39	179 (51.2)
	40–49	73 (20.7)
	>50	31 (9.1)
Gender	Male	122 (35)
	Female	227 (65)
Marital status	Single	160 (46)
	Married	189 (54)
Work experience (years)	1–5	155 (44.2)
	6–10	61 (17.9)
	11–15	60 (17.2)
	Above 15	73 (20.7)

**Table 3:** Measurement model evaluation.

Latent variables	Factor loadings	CR	AVE
*Person-job fit*			
PJF1	0.825	0.837	0.632
PJF2	0.804		
PJF3	0.755		

*Person-organization fit*			
POF1	0.783	0.855	0.598
POF2	0.679		
POF3	0.790		
POF4	0.832		

*Person-supervisor fit*			
PSF2	0.522	0.733	0.599
PSF3	0.962		

*Job satisfaction*			
JS1	0.571	0.909	0.628
JS2	0.815		
JS3	0.858		
JS4	0.845		
JS5	0.854		
JS6	0.771		

*Psychological empowerment*			
PE1	0.801	0.943	0.624
PE10	0.775		
PE2	0.758		
PE3	0.808		
PE4	0.778		
PE5	0.789		
PE6	0.818		
PE7	0.756		
PE8	0.830		
PE9	0.783		

*Turnover intention*			
TI1	0.834	0.907	0.621
TI2	0.827		
TI3	0.738		
TI4	0.762		
TI5	0.776		
TI6	0.785		

*Note.* CR: composite reliability; AVE: average variance extracted. PSF1, PE11, and PE12 are deleted due to low factor loadings.

**Table 4:** Heterotrait-monotrait (HTMT) ratio.

	JS	PE	PJF	POF	PSF	TI
JS						
PE	0.829					
PJF	0.213	0.17				
POF	0.175	0.195	0.159			
PSF	0.604	0.758	0.136	0.702		
TI	0.817	0.738	0.167	0.124	0.454	

*Note.* JS: job satisfaction; PE: psychological empowerment; PJF: person-job fit; POF: person-organization fit; PSF: person-supervisor fit; TI: turnover intention.

**Table 5:** Path coefficients and hypotheses testing.

Hypotheses	Direct relationships	Path coefficient	*t*-value	*p* value	Results
H1	P-J fit - > JS	0.132	2.743	0.006^*∗*^	Supported
H2	P-O fit - > JS	0.022	0.467	0.641	Not supported
H3	P-S fit - > JS	0.423	8.180	0.000^*∗∗∗*^	Supported
H4	JS - > TI	0.577	6.234	0.000^*∗∗∗*^	Supported
H5	PE^*∗*^JS - > TI	0.570	2.478	0.013^*∗*^	Supported

*Note.* ^*∗*^ *p* < 0.05; ^*∗∗∗*^*p* < 0.001 (one-tailed).

**Table 6:** Mediating effect of job satisfaction.

Hypotheses	Specific indirect relationships	Path coefficient	*t*-value	*p* value	Results
H6	P-J fit - > JS - > TI	0.076	2.38	0.018^*∗*^	Supported
H7	P-O fit - > JS - > TI	0.013	0.448	0.654	Not supported
H8	P-S fit - > JS- > TI	0.244	5.333	0.000^*∗∗∗*^	Supported

*Note.* ^*∗*^ *p* < 0.05; ^*∗∗∗*^*p* < 0.001 (two-tailed).

## Data Availability

The underlying data from the results of this study reside with the corresponding author.
